# High TNFRSF12A level associated with MMP-9 overexpression is linked to poor prognosis in breast cancer: Gene set enrichment analysis and validation in large-scale cohorts

**DOI:** 10.1371/journal.pone.0202113

**Published:** 2018-08-24

**Authors:** Jungho Yang, Kyueng-Whan Min, Dong-Hoon Kim, Byoung Kwan Son, Kyoung Min Moon, Young Chan Wi, Seong Sik Bang, Young Ha Oh, Sung-Im Do, Seoung Wan Chae, Sukjoong Oh, Young Hwan Kim, Mi Jung Kwon

**Affiliations:** 1 Departments of Pathology, Kangbuk Samsung Hospital, Sungkyunkwan University School of Medicine, Seoul, Republic of Korea; 2 Department of Pathology, Hanyang University Guri Hospital, Hanyang University College of Medicine, Guri, Gyeonggi-do, Republic of Korea; 3 Department of Internal Medicine, Eulji Hospital, Eulji University School of Medicine, Seoul, Republic of Korea; 4 Department of Internal Medicine, Gangneung Asan Hospital, University of Ulsan College of Medicine, Gangneung, Republic of Korea; 5 Department of Pathology, Hanyang University College of Medicine, Seoul, Republic of Korea; 6 Departments of Internal Medicine, Kangbuk Samsung Hospital, Sungkyunkwan University School of Medicine, Seoul, Republic of Korea; 7 Departments of Nuclear Medicine, Kangbuk Samsung Hospital, Sungkyunkwan University School of Medicine, Seoul, Republic of Korea; 8 Department of Pathology, Hallym University Sacred Heart Hospital, Hallym University College of Medicine, Anyang, Gyeonggi-do, Republic of Korea; University of South Alabama Mitchell Cancer Institute, UNITED STATES

## Abstract

**Background:**

Matrix metalloproteinase-9 (MMP-9) is associated with remodelling of the extracellular matrix and invasion in various cancers. Identifying proteins connected to high MMP-9 expression is important in explaining its mechanisms. Our study aims to shed light on genes associated with high MMP-9 expression and to discuss their clinical impact in breast cancer.

**Methods:**

We evaluated 173 breast cancer cases from the Kangbuk Samsung Hospital, with 1964 cases from the Molecular Taxonomy of Breast Cancer International Consortium database serving as a validation cohort. We investigated relationships between MMP-9 expression and clinicopathological characteristics. We then used gene set enrichment analyses to detect the association of genes with MMP-9 overexpression, and performed survival analyses to determine the significance of the gene in three independent cohorts.

**Results:**

High MMP-9 expression correlated with poor prognosis in univariate and multivariate analyses. Using gene set enrichment analysis, we found that tumour necrosis factor receptor superfamily member 12A (TNFRSF12A) was linked to high MMP-9 expression. In the survival analysis of three published data sets (METABRIC, GSE1456, GSE20685), high TNFRSF12A was relevant to a poor survival rate.

**Conclusions:**

High levels of TNFRSF12A associated with MMP-9 overexpression may be important to explain the progression of breast cancer, and survival could be improved using therapy targeting TNFRSF12A.

## Introduction

Breast cancer is one of the most common types of cancer diagnosed and main cause of death from cancer among women (23% of all cancer cases, 14% of deaths from cancer) [1]. Invasive ductal carcinoma (IDC) represents 70 to 80 percent of all breast cancer cases, followed by lobular carcinoma, tubular/cribriform carcinoma, mucinous carcinoma, medullary carcinoma, and papillary carcinoma [2]. Several findings, such as cancer development, cancer stage, oestrogen receptor (ER) and progesterone receptor (PR) expression, human epithelial growth factor receptor 2 (*HER2*) gene amplification, p53 overexpression, and *BRCA1* and *BRCA2* mutations, are taken into account in the development, treatment, and management of breast cancer [3]. Recently, in molecular subtyping using the above markers, breast cancer was classified into five major subtypes: luminal A, luminal B HER2-negative, luminal B HER2-positive, basal-like, and HER2-positive [4].

Matrix metalloproteinases (MMPs) is critical in decomposition of the extracellular membrane (ECM), including the basement membrane, a specialized matrix comprising type IV collagen, laminin, entactin, proteoglycans, and glycosaminoglycans [5]. Twenty-five MMPs have been identified to play critical roles in cancer progression via many carcinogenic processes, including remodelling of the microenvironment. Recently, studies on treatments targeting MMP-2 have also been reported [6,7].

MMP-9, a member of the MMPs, has long been postulated to play an important role in poor prognosis in tumour cells [8]. MMP-9 causes the release of the biologically active form of vascular endothelial growth factor, which is critical in angiogenesis [9,10]. The process is completed by the direct proteolytic degradation of vascular basement membrane proteins, meaning that MMP-9 can be essential in the creation of new blood vessels [10]. MMP-9 degrades the collagen-rich ECM, which has been related to cancer invasiveness, metastasis, and recurrence [11,12]. Therefore, MMP-9 has been considered an important prognostic predictor in various tumours.

In previous studies, MMP-9 was reported to be overexpressed in aggressive tumours [13–15]. Additionally, MMP-9 is required for endothelial cell migration and tube formation, and is likely to be important in cerebral angiogenesis [16]. Thus, an increase in MMP-9 levels associated with vascular angiogenesis could promote tumour invasion and metastasis. MMP-9 is involved in a variety of developmental processes because of its important role in angiogenesis. MMP-9 deficiency leads to bone development disorders, particularly bone formation delay caused by insufficient angiogenesis in the growth plate [17]. Recent studies demonstrated that MMP-9 could be involved in natural killer (NK) cell dysfunction by promoting tumour immune avoidance. Hence, the effect of NK cell-based immunotherapy could be enhanced by controlling MMP-9 activity [18].

**Fig 1:**
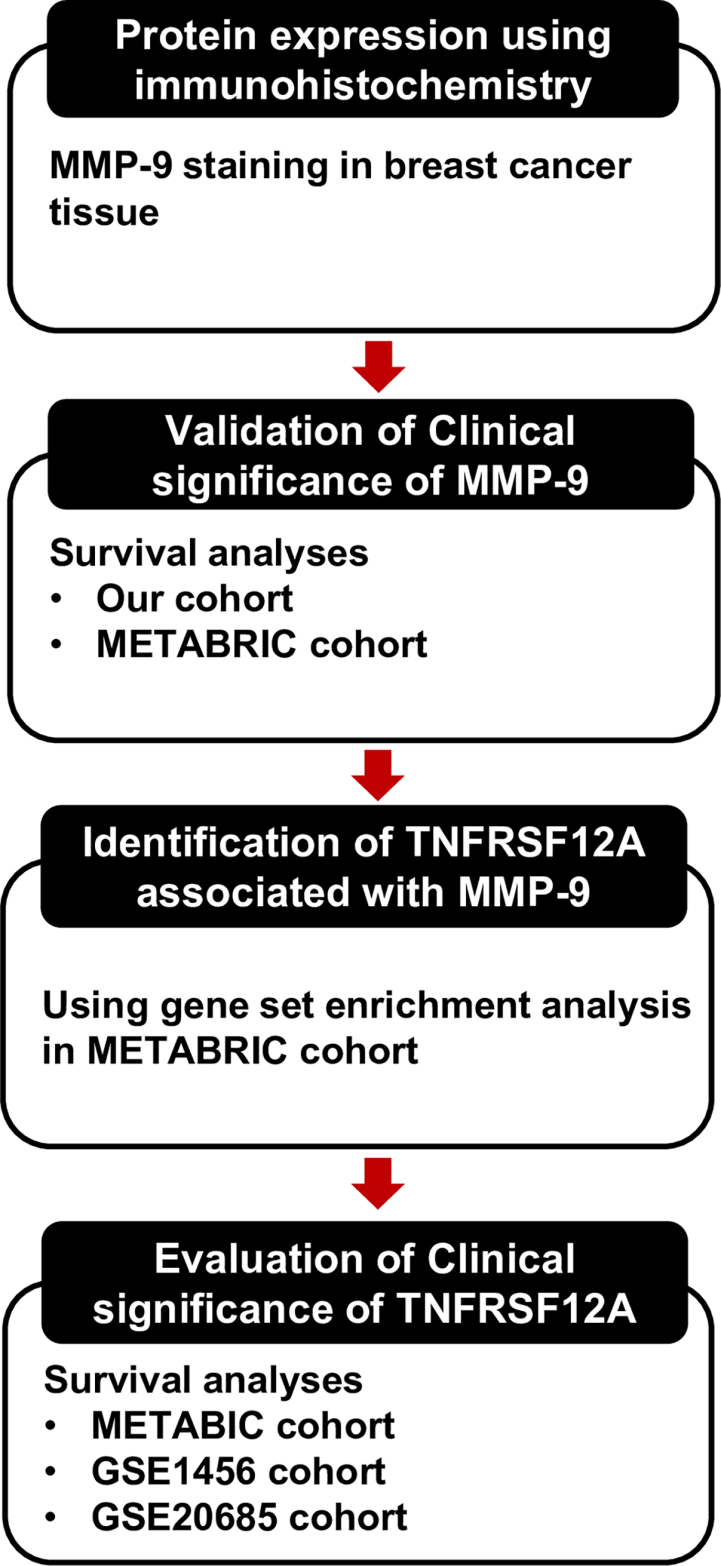
A schematic diagram depicting the analysis pipeline in our study.

The present study aimed to assess whether MMP-9 levels could predict patient survival and to analyse its prognostic value in comparison with the Molecular Taxonomy of Breast Cancer International Consortium (METABRIC) database. We further aimed to investigate the prognostic utility of MMP-9 in two cohorts (our data and METABRIC data), and to identify genes associated with MMP-9 overexpression using gene set enrichment analysis (GSEA). In addition, survival analysis was performed to confirm the clinical significance of the newly identified genes (**Fig 1**).

## Materials and methods

### Patient selection

This study included patients who were treated with breast cancer at Kangbuk Samsung Medical Center in Korea between 2000 and 2005. In randomly selected patients, immunohistochemical staining was performed on 173 specimens obtained from surgical treatment. The Reporting Recommendations for Tumour Marker Prognostic Studies (REMARK) criteria were followed throughout this study. The inclusion criteria were: 1) patients with histopathological evidence of primary invasive ductal carcinoma confirmed by pathologists, and known clinical outcome; and 2) patients without preoperative concurrent chemoradiotherapy.

The mean age of the patients was 47 (range: 25–79) years. Treatments comprised 163 (94.2%) modified radical mastectomies with axillary lymph node dissection and 10 (5.8%) breast-conserving surgeries. At the time of surgery, the T and N stages, according to the 8^th^ American Joint Committee on Cancer (AJCC), were distributed as follows: T1, 61 (35.3%); T2, 100 (57.8%); T3, 11 (6.4%), T4, 1 (0.6%); N0, 68 (39.3%); N1, 62 (35.8%); N2, 23 (13.3%); and N3, 20 (11.6%). The distribution of AJCC stages was as follows: stage I, 31 (17.9%); stage II, 97 (56.1%); and stage III, 45 (26%).

Haematoxylin and eosin (H&E)-stained slides were reviewed by at least two pathologists for each case (Min and Kim). The tumours were characterized according to their clinicopathological features, such as size, margins, number of tumours (single or multiple), histological grade, necrosis, and tumour fibrosis. Cases with unavailable paraffin blocks and inadequate clinical history were excluded. The histological grade was determined according to the modified Bloom–Richardson–Elston grading system [19].

This study (involving human participants) was approved by the Ethics Committee of the Kangbuk Samsung Hospital, Seoul, Republic of Korea (KBSMC 2018-02-026) and performed according to the ethical standards of the Declaration of Helsinki, as revised in 2008. The review conducted by our institutional review board confirmed that informed consent was not necessary for this study.

### Tissue microarray construction and immunohistochemistry

The most morphologically representative and non-necrotic areas were carefully selected and marked on the H&E-stained slides. The tissue microarray (TMA) specimens were assembled using a tissue-array instrument (AccuMax Array; ISU ABXIS Co. Ltd, Seoul, Korea) consisting of thin-walled stainless steel punches and stylets for emptying and transferring the needle’s content. The assembly was held in an X-Y position guide equipped with semiautomatic micrometres, with a 1-mm increment between the individual samples and a 4-mm punch depth stop device. The instrument was briefly used to create holes in a recipient block with defined array cores. The micro-needle was used to transfer the tissue cores into the recipient block. Taking into account the limitations of the representative areas of the tumour, we used duplicate 3-mm-diameter tissue cores from each donor block. The percentage of tumour in the tissue cores was greater than 70%.

Serial sections, 4-μm each, were cut from the array blocks and deparaffinised using routine techniques. Immunostaining for PR, ER, HER2, and marker of proliferation Ki-67 (Ki-67) was performed using the Dako Autostainer Universal Staining System (DakoCytomation, Carpinteria, CA, USA), and the ChemMate DAKO EnVision Detection Kit. The following primary antibodies were used: anti-ER (1:200; Lab Vision Corporation, Fremont, CA, USA), anti-PR (1:200; Dako, Glostrup, Denmark), anti-HER2 (1:200; Dako), and anti-Ki-67 (1:200; Dako). Immunostaining was used to classify the five molecular subtypes of breast cancer: luminal A (ER-positive and/or PR-positive; HER2-negative; Ki-67 low), luminal B HER2-negative (ER-positive and/or PR-positive; HER2-negative; Ki-67 high), luminal B HER2-positive (ER-positive and/or PR-positive; HER2-positive), HER2-positive (ER-negative and PR-negative; HER2-positive), and triple-negative (ER-negative and PR-negative; HER2-negative) [4].

**Fig 2:**
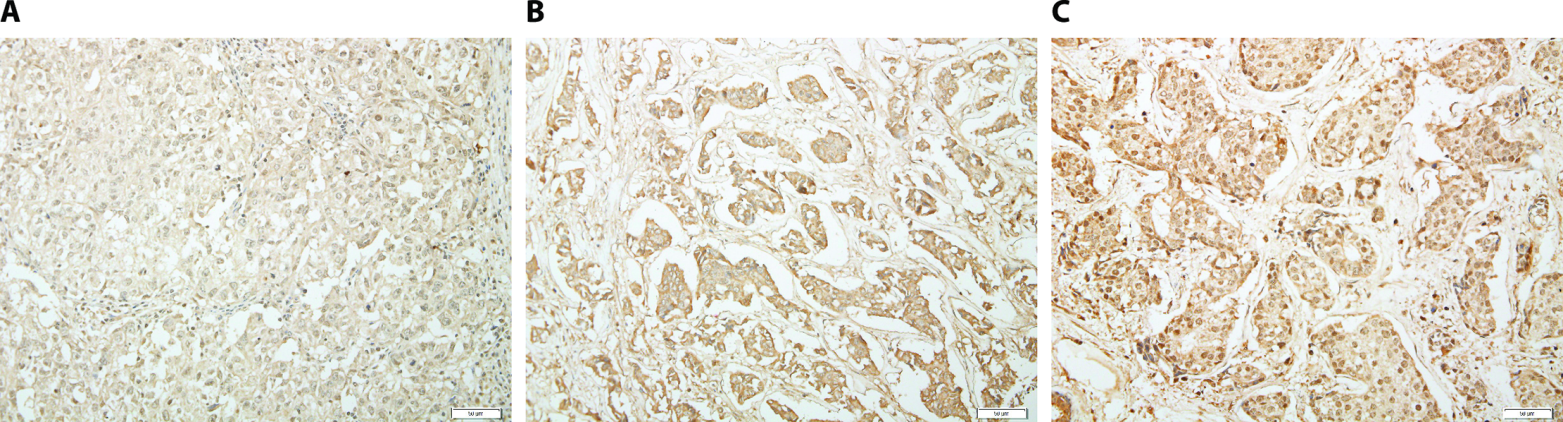
Representative photomicrographs showing MMP-9 expression of weak (A), moderate (B), and strong (C) intensity by immunohistochemical staining in invasive ductal carcinoma (original magnification, ×400).

For MMP-9, affinity-purified rabbit anti-human MMP-9 polyclonal antibodies (1:100; NeoMarkers Corporation, Fremont, CA, USA) were used, and detection was performed using the Ultra Tech HRP Streptavidin-Biotin Detection System (Beckman Coulter, Marseille, France) with an automatic staining machine (Bond Intense Detection Kit; Leica Biosystems, Newcastle, UK). Positive MMP-9 immunostaining was defined as exclusively cytoplasmic staining without nuclear staining, and was graded according to the intensity of staining of tumour cells. Staining intensity was scored on a scale of 0 to 3 (0 = negative; 1 = weak; 2 = moderate; 3 = strong) (**Fig 2**). MMP-9 expression was determined as either negative (intensity ≤ 1) or positive (intensity > 1) using the receiver operating characteristic (ROC) curve.

### Data extraction and GSEA from the METABRIC database

Gene data (cDNA microarray profiling, Illumina HT-12 v3 platform) from METABRIC were downloaded from the domain training data (www.cbioportal.org/) [20]. *MMP9* expression and clinicopathological data (AJCC, tumour size, histological grade, ER, PR, HER2, and overall survival) were extracted using the R package (http://www.r-project.org/). Normal samples as well as tumour samples with missing data were excluded from analysis.

GSEA is a method of analysing and interpreting microarray and other such data based on biological information. These biological sets can be published information about a biochemical pathway or coexpression obtained in a previous experiment. GSEA was performed using GSEA version 3.0 from the Broad Institute at MIT (software.broadinstitute.org/gsea/index.jsp/).

The parameters used for the analysis were as follows. The data set had 17,786 features in the Molecular Signatures Database (MSigDB). To identify the oncogenic signatures that are significantly enriched in genes associated with high *MMP9* expression, a gene set database (c6.all.v6.1symbols.gmt including 189 gene sets) was used for GSEA, with 1000 permutations used to calculate the p values, and permutation type was set to phenotype. We defined a meaningful gene set as one with a false discovery rate (FDR) of < 0.2, a family wise-error rate (FWER) of < 0.4, and *p* < 0.05.

Gene expression profiling data sets such as GSE1456 and GSE20685 were downloaded from the GEO database (http://www.ncbi.nlm.nih.gov/geo/). In three independent cohorts, including METABRIC, GSE1456, and GSE20685, survival analyses were performed to verify the clinical significance of meaningful genes identified using GSEA.

### Statistical analysis

Correlations between clinicopathological parameters and MMP-9 expression were analysed using the χ^2^ test and a linear-by-linear association test. Disease-free survival (DFS) was defined as survival from the date of diagnosis to recurrence/new distant metastasis, and overall survival (OS) was defined as survival from the date of diagnosis to cancer-related death. Survival curves were generated using the Kaplan–Meier method, applying the cut-off values calculated from the ROC, and then compared using the log rank test. Multivariate analysis was performed to identify independent prognostic markers for DFS and OS using a Cox multistep regression model. A two-tailed *p* value of < 0.05 was considered statistically significant. All data were analysed using SPSS statistics for Windows version 24.0 (SPSS Inc., Chicago, IL, USA) or the R package (http://www.r-project.org/).

## Results

### Clinicopathological parameters according to MMP-9 expression

**Fig 3:**
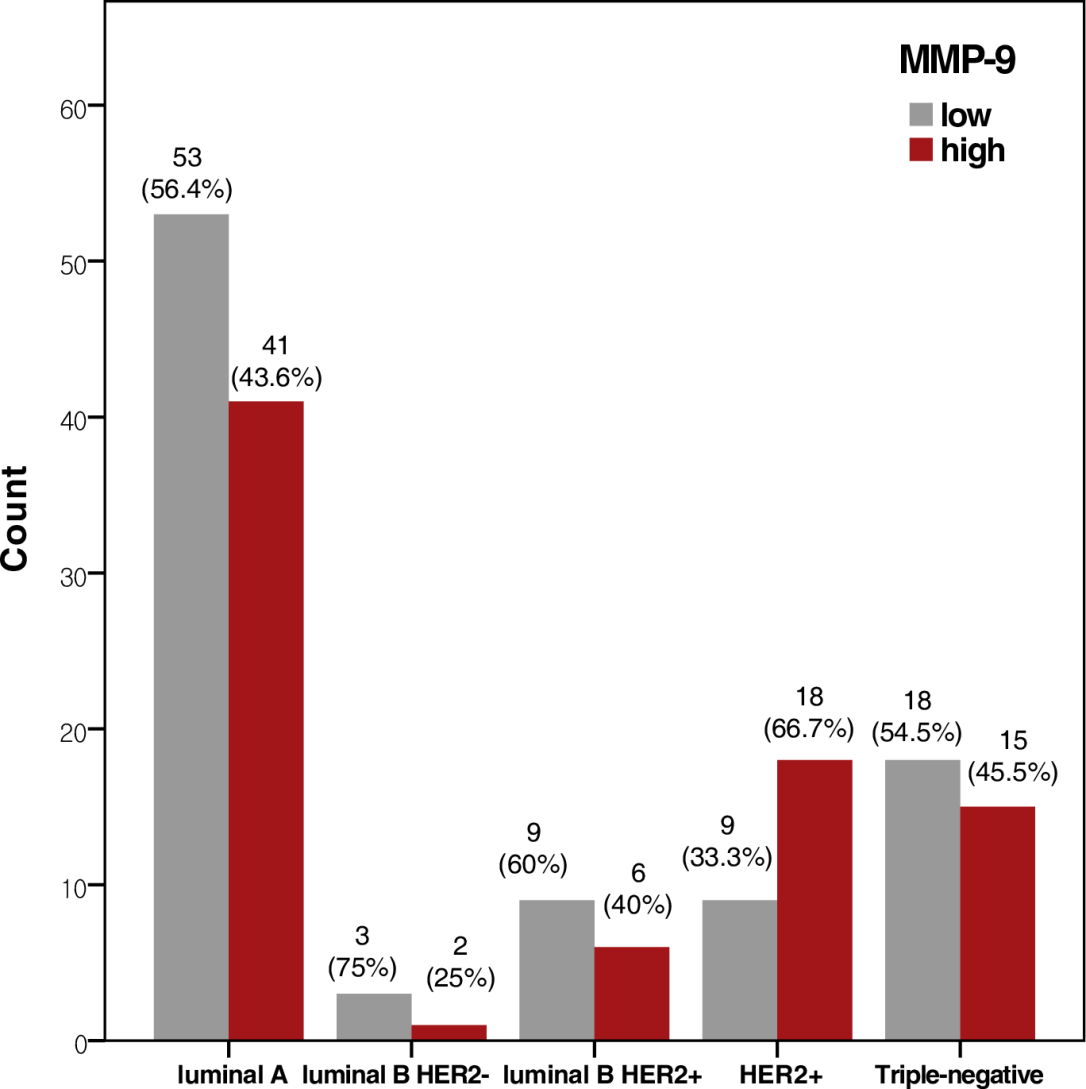
Expression of MMP-9 according to molecular subtype. High MMP-9 expression was more frequently observed in the HER2 subtype than in the other subtypes, including luminal A, luminal B HER2-negative, luminal B HER2-positive, HER2-positive, and triple-negative subtype (*p* = 0.024).

In the breast cancer cases from our hospital, high MMP-9 expression was significantly associated with high histological grade. In the METABRIC cases, *high MMP-9 expression* was significantly associated with high histological grade, ER/PR negativity, and HER2 positivity (all *p* < 0.001) (**Table 1**). In both datasets, high MMP-9 expression was more frequently observed in patients with advanced AJCC stage. However, the relationships were not statistically significant (*p* = 0.080 and 0.473 for our data and the METABRIC data, respectively). High MMP-9 expression was more frequently observed in the HER2 subtype than in the luminal A/B or triple-negative subtype (*p* = 0.024) (**Table 2**, **Fig 3**).

**Table 1:** Clinicopathological parameters of MMP-9 from our data and METABRIC data.

Parameter	MMP-9 (our case)		*p* value	MMP-9 (METABRIC)		*p* value
	Low (n = 92), n (%)	High (n = 81), n (%)		Low (n = 235), n (%)	High (n = 1118), n (%)	
AJCC						
I	13 (14.1)	18 (22.2)	0.080^a^	85 (36.2)	370 (33.1)	0.473^a^
II	55 (59.8)	42 (51.9)		132 (56.2)	646 (57.8)	
III	24 (26.1)	21 (25.9)		16 (6.8)	95 (8.5)	
IV	-	-		2 (0.9)	7 (0.6)	
Tumour size						
≤2 cm	34 (37.0)	28 (34.6)	0.744	109 (46.4)	499 (44.6)	0.624
>2 cm	58 (63.0)	53 (65.4)		126 (53.6)	619 (55.4)	
Histological grade						
1	14 (15.2)	12 (14.8)	**0.025**^**b**^	36 (15.3)	78 (7)	**<0.001**^**b**^
2	49 (53.3)	30 (37.0)		132 (56.2)	401 (35.9)	
3	29 (31.5)	39 (48.1)		67 (28.5)	639 (57.2)	
Lymphatic invasion						
Negative	46 (50.0)	32 (39.5)	0.166	-	-	-
Positive	46 (50.0)	49 (60.5)		-	-	
Perineural invasion						
Negative	79 (85.9)	70 (86.4)	0.917	-	-	-
Positive	13 (14.1)	11 (13.6)		-	-	
Tumour necrosis						
Absence	49 (5.3)	45 (55.6)	0.762	-	-	-
Presence	43 (46.7)	36 (44.4)		-	-	
ER						
Negative	27 (29.3)	33 (40.7)	0.116	14 (6)	299 (26.7)	**<0.001**
Positive	65 (70.7)	48 (59.3)		221 (94)	819 (73.3)	
PR						
Negative	41 (44.6)	43 (53.1)	0.263	72 (30.6)	577 (51.6)	**<0.001**
Positive	51 (55.4)	38 (46.90		163 (69.4)	541 (48.4)	
HER2						
Negative	71 (77.2)	55 (67.9)	0.171	221 (94)	964 (86.2)	**0.001**
Positive	21 (22.8)	26 (32.1)		14 (6.0)	154 (13.8)	

AJCC, American Joint Committee on Cancer, 8^th^ edition; ER, oestrogen receptor; HER2, human epidermal growth factor receptor 2; METABRIC, Molecular Taxonomy of Breast Cancer International Consortium; MMP, matrix metalloproteinase; PR, progesterone receptor.

^a^AJCC stage I/II versus III.

^b^Histological grade 1/2 versus 3.

Bold indicates *p* < 0.05.

**Table 2:** MMP-9 expression according to molecular subtype.

	Luminal A	Luminal B HER2-	Luminal B HER2+	HER2	Triple-negative	*p* value
MMP-9						
Low	53 (56.4)	3 (75)	9 (60)	9 (33.3)	18 (54.5)	**0.024**^a^
High	41 (43.6)	1 (25)	6 (40)	18 (66.7)	15 (45.5)	

HER2, human epidermal growth factor receptor 2; MMP, matrix metalloproteinase.

^a^Comparison of MMP-9 expression between HER2 and other subtypes.

Bold indicates *p* < 0.05.

### Survival analyses according to MMP-9 expression

**Fig 4:**
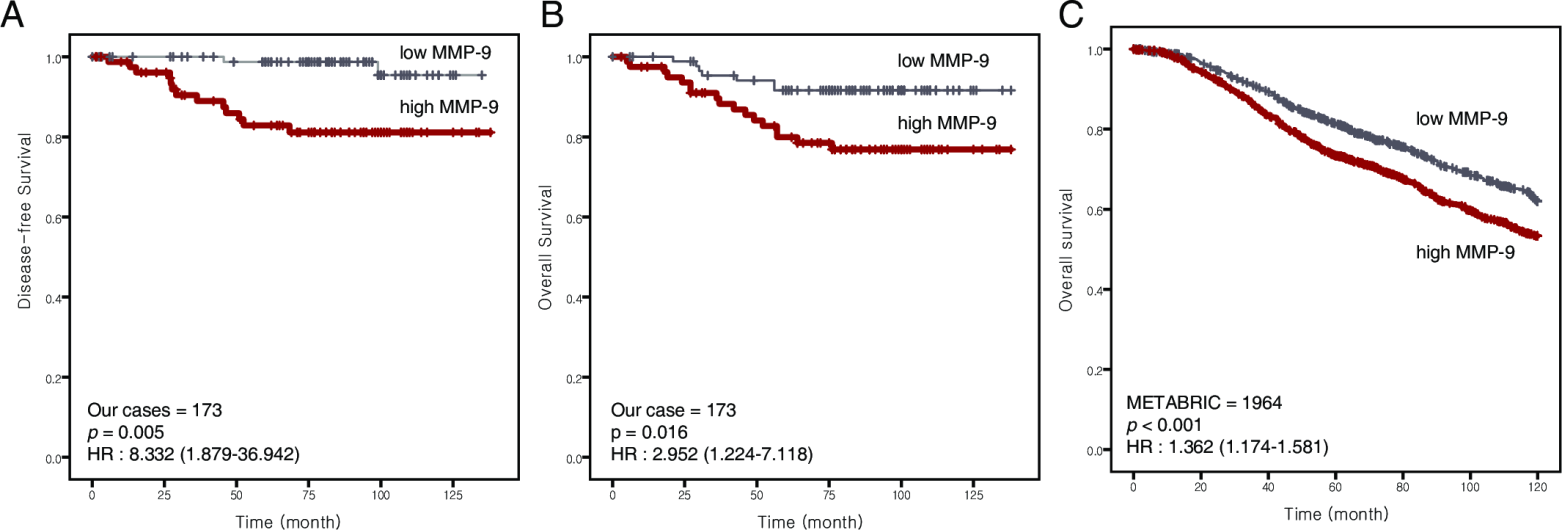
Survival curves derived using the Kaplan–Meier method showing correlation with MMP-9. (A) Disease-free survival, (B) overall survival, (C) overall survival in the METABRIC data (*p* = 0.001, 0.011, and 0.002, respectively).

In our data, high MMP-9 expression was significantly correlated with poor DFS and OS (*p* = 0.001 and 0.001, respectively). After adjusting for confounders, including T and N stage, histological grade, lymphatic/perineural invasion, ER/PR and HER2, there was still a significant relationship between MMP-9 and DFS and OS (*p* = 0.004 and 0.011, respectively). In the METABRIC data, *high MMP-9 expression* was significantly associated with worse OS in univariate analysis (*p* = 0.004). After adjusting for confounders, including AJCC stage, histological grade, ER/PR and HER2, the association between *high MMP-9 expression* and worse OS remained significant (*p* = 0.027) (**Table 3, Fig 4**).

**Table 3:** Disease-free and overall survival analyses according to MMP-9 levels.

Survival	Univariate^a^	Multivariate ^b^	HR	95% CI	
Disease-free survival					
MMP-9 (low *vs*. high)	**0.001**	0.004	10.901	2.124	55.950
T stage (1 or 2 *vs*. 3)	**0.649**	0.201	4.477	0.449	44.641
N stage (0, 1 or 2 *vs*. 3)	**0.147**	0.122	3.393	0.720	15.989
Histologic grade (1 or 2 *vs*. 3)	**0.039**	0.615	1.429	0.356	5.740
Lymphatic invasion (negative *vs*. positive)	0.515	0.198	0.407	0.104	1.598
Perineural invasion (negative vs. positive)	**0.013**	0.023	4.181	1.215	14.386
ER/PR (positive vs. negative)	**0.05**	0.342	1.985	0.483	8.164
HER2 (negative vs. positive)	0.105	0.354	1.803	0.519	6.263
Overall survival					
MMP-9 (low vs. high)	**0.011**	0.011	3.720	1.354	10.221
T stage (1 or 2 *vs*. 3)	**<0.001**	0.002	5.894	1.926	18.034
N stage (0, 1 or 2 *vs*. 3)	**<0.001**	0.002	6.047	1.975	18.516
Histologic grade (1 or 2 *vs*. 3)	**<0.001**	0.690	1.273	0.389	4.170
Lymphatic invasion (negative *vs*. positive)	<0.001	0.276	2.125	0.547	8.254
Perineural invasion (negative *vs*. positive)	0.015	0.106	2.203	0.845	5.744
ER/PR (positive vs. negative)	**0.003**	0.364	1.693	0.543	5.277
HER2 (negative vs. positive)	**<0.001**	0.016	3.487	1.264	9.617

T and N stages are according to the American Joint Committee on Cancer, 8^th^ edition.

ER/PR, estrogen receptor/progesterone receptor; HER2, human epithelial growth factor receptor 2

^a^Log rank test.

^b^Cox proportional hazards model.

Bold indicates *p* < 0.05.

### Identification of the association of *TNFRSF12A* associated with *high MMP-9 expression*

**Fig 5:**
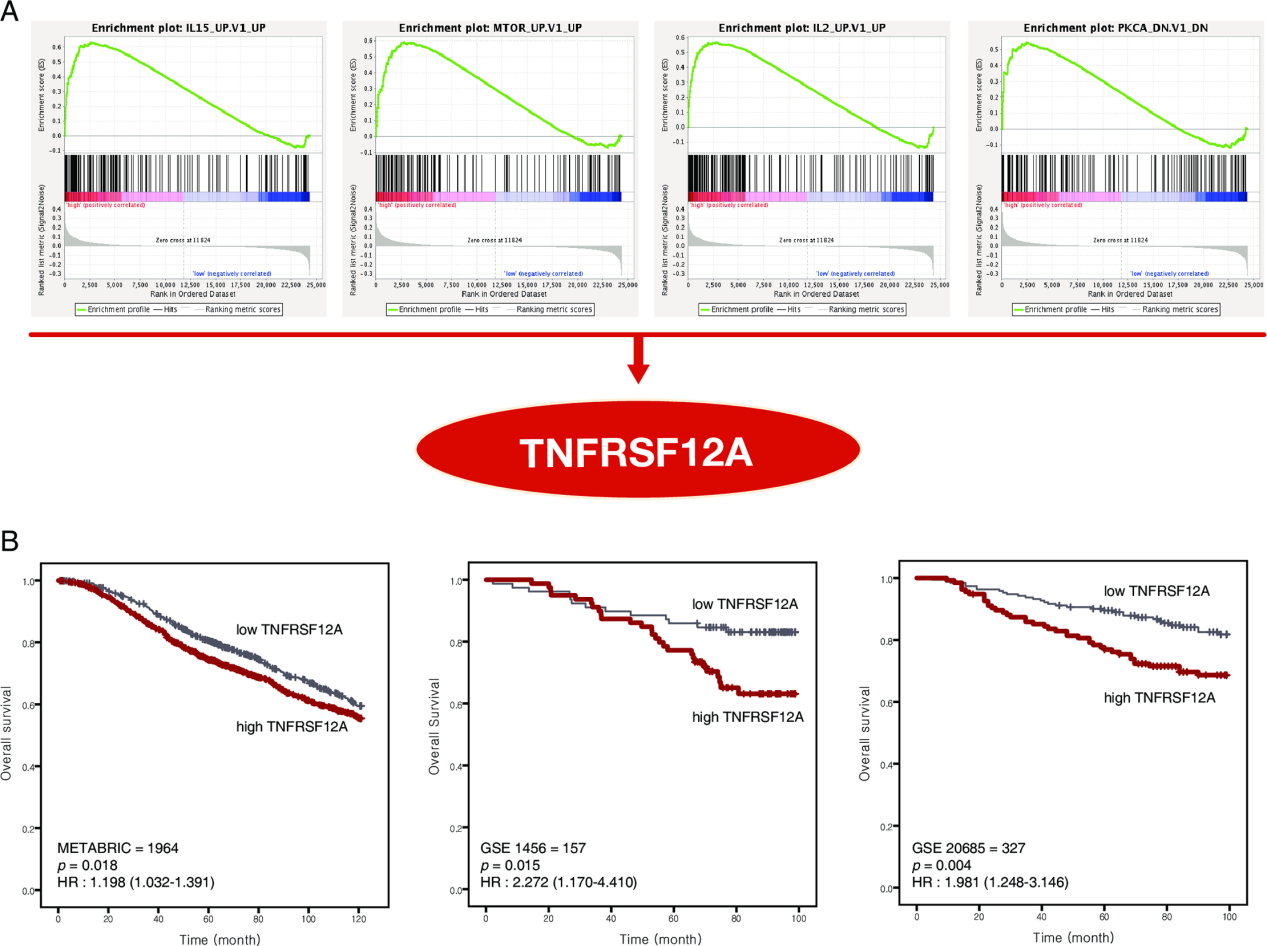
Gene expression data analysed using gene set enrichment analysis (GSEA) to extract biological information. In each thumbnail, the green curve represents the evolution of the density of the genes identified in the RNA-seq. GSEA calculates these by walking down the ranked-ordered list of genes, increasing a running-sum statistic when a gene is in the gene set and decreasing it when it is not. (A) *TNFRSF12A* was found commonly in four gene sets (IL15_UP.V1_UP, MTOR_UP.V1_UP, IL2_UP.V1_UP and PKCA_DN.V1_DN) with high MMP-9 expression. (B) High TNFRSF12A was associated with poor prognosis in three independent cohorts (METABRIC, GSE1456, and GSE20685).

We conducted GSEA to identify the genes associated with high MMP-9 expression and found eleven significantly enriched gene sets: RB_P107_DN.V1_UP, IL15_UP.V1_UP, ATF2_S_UP.V1_UP, ATF2_UP.V1_UP, CSR_LATE_UP.V1_UP, P53_DN.V2_UP, E2F1_UP.V1_UP, MTOR_UP.V1_UP, IL2_UP.V1_UP, PKCA_DN.V1_DN, and BMI1_DN_MEL18_DN.V1_UP (**Table 4**). We found that *TNFRSF12A* (encoding tumour necrosis factor receptor superfamily member 12A) was common in four gene sets (IL15_UP.V1_UP, MTOR_UP.V1_UP, IL2_UP.V1_UP and PKCA_DN.V1_DN). Survival analyses were performed in three data sets (METABRIC, GSE1456, GSE20685) to determine the significance of *TNFRSF12A* expression levels. High *TNFRSF12A* expression was significantly associated with worse OS (all *p* < 0.05) (**Fig 5)**.

**Table 4:** Gene sets related to high MMP-9 expression.

NAME	SIZE	ES	NES	NOM (p-value)	FDR (q-value)	FWER (p-value)
RB_P107_DN.V1_UP	134	0.667521	1.7672	<0.000001	0.012797	0.006
IL15_UP.V1_UP	166	0.632059	1.738388	<0.000001	0.007296	0.007
ATF2_S_UP.V1_UP	175	0.571068	1.715975	<0.000001	0.00982	0.014
ATF2_UP.V1_UP	170	0.566506	1.700243	<0.000001	0.008313	0.017
CSR_LATE_UP.V1_UP	162	0.645542	1.675572	0.003984	0.009363	0.026
P53_DN.V2_UP	137	0.572954	1.674256	<0.000001	0.008579	0.028
E2F1_UP.V1_UP	179	0.559687	1.645125	0.001949	0.01346	0.053
MTOR_UP.V1_UP	150	0.59151	1.632898	<0.000001	0.016188	0.068
IL2_UP.V1_UP	168	0.570225	1.604615	<0.000001	0.018264	0.096
PKCA_DN.V1_DN	151	0.547922	1.59843	<0.000001	0.018233	0.105
BMI1_DN_MEL18_DN.V1_UP	133	0.627022	1.551241	0.002004	0.019859	0.185

ES, Enrichment Score; NES, Normalized Enrichment Score; FDR, False Discovery Rate; NOM, Nominal p Value; FDR, False discovery rate; FWER, Family wise-error rate

## Discussion

In cancer processes, the activities of certain extracellular matrix degradative enzymes provide favourable conditions for tumour growth and subsequent dissemination and metastasis. This study evaluated MMP-9 expression in relation to various clinicopathological parameters in breast cancer. Interestingly, our results revealed that high MMP-9 expression is associated with a high histological grade and HER2 subtype, as well as worse DFS and OS in patients with breast cancer. To further reinforce the implications of these findings, we reanalysed the association of MMP-9 with clinical data from METABRIC, a large-scale study, to improve the reproducibility of the findings. As in our study, high MMP-9 expression was associated with poor clinical outcomes, such as high histological grade and lower OS. In addition, MMP-9 was significantly associated with the levels of ER, PR, and HER2, which are important factors in breast cancer treatment. Thus, MMP-9 might play a major role in promoting cancer progression, and the evaluation of enzymes like MMP-9 may be helpful in predicting clinical outcomes.

Many published studies have demonstrated that MMP-9 overexpression is associated with various malignant tumours [12,21,22]; however, its significance was not clear in other reports [23,24]. A study by Scorilas et al. demonstrated that MMP-9 overexpression is associated with a favourable prognosis in patients with breast cancer without lymph node metastasis [25]. A study by Pellikainen et al. suggested that MMP-9 expression in stromal cells is associated with poor prognosis in patients receiving hormonal therapy; however, MMP-9 expression in tumour cells indicated good prognosis [24]. Therefore, the patient’s prognosis differs depending on the specific state of the individual patient and whether MMP-9 expression occurs in the tumour or stromal cells. Therefore, controversy still exists regarding the relationship between MMP-9 expression and clinical outcomes in various malignant tumours. The difference in the prognostic value of MMP-9 expression in stromal and tumour cells might reflect the presence of active and inactive forms of MMP-9. Generally, MMPs are secreted as inactive proenzymes that are activated by many mechanisms, including organomercurials, chaotropic agents, and other proteases. The amount of the active form of MMP-9 in stromal cells and tumour cells may differ, which could account for the differences in clinical outcomes.

In our data, the expression of MMP-9 was significantly correlated with unfavourable clinicopathological parameters, even though its expression was unrelated to other parameters. Therefore, MMP-9 expression in tumour cells might play an important role in several prognostic processes. High MMP-9 expression was associated with poor OS, and the association remained significant after adjustment for potential confounders. Notably, our results, showing that high MMP-9 expression was associated with poor prognosis, were validated in a large-scale breast cancer cohort, METABRIC. Nevertheless, a dissociation between survival and MMP-9 is thought to derive from incredible advance of technical surgical procedures, chemotherapy, hormonal therapy, and the number of immune cells raised against tumour cells. In addition, the following factors can be considered as variables that can lead to conflicting results: study design, cancer type, ethnic factors, and the number of patients.

We identified *TNFRSF12A* as an MMP-9-related gene in patients with breast cancer. TNFRSF12A is one of the TNF ligand family that induces angiogenesis *in vivo* [26]. In the GEO database, the survival rate of patients in association with *TNFRSF12A* levels was analysed to determine its clinical significance. High levels of *TNFRSF12A* accompanied by MMP-9 overexpression were associated with poor OS. This result suggested that high *TNFRSF12A* expression is related to aggressive behaviour of breast cancer. The relationship between the MMP-9 and TNFRSF12A needs to be proven through experimentation; however, we speculated that TNFRSF12A associated with high MMP-9 expression might accelerate cancer progression through increased angiogenesis.

This study had some limitations that should be acknowledged. First, because our cross-sectional study did not show sustained relationships over time, as prospective studies do, it is difficult to come to a definitive conclusion. Further *in vitro* and/or *in vivo* studies of MMP-9 expression may be necessary. Second, representative tissue may not have been assessed because it was included in only two sections of each tumour specimen. Third, alterations in MMP-9 expression could not be evaluated, because the level of MMP-9 expression before and after adjuvant therapy was not investigated.

## Conclusions

In summary, the study showed that high MMP-9 expression is significantly associated with poor DFS and OS in two cohorts (our data and METABRIC data). High expression of *TNFRSF12A*, a gene associated with MMP-9 overexpression through GSEA, was shown to be associated with worse OS in three independent genetic data sets. The newly discovered gene, *TNFRSF12A*, could be an important factor in explaining breast cancer progression associated with MMP-9. In the future, targeted therapy for TNFRSF12A might improve the survival of patients with MMP-9 overexpression.
